# Professional help-seeking behaviour for mental health problems among veterinarians in Norway: a nationwide, cross-sectional study (The NORVET study)

**DOI:** 10.1186/s12889-022-13710-y

**Published:** 2022-07-07

**Authors:** Helene Seljenes Dalum, Reidar Tyssen, Torbjørn Moum, Magne Thoresen, Erlend Hem

**Affiliations:** 1 Department of Behavioural Medicine, Institute of Basic Medical Sciences, Faculty of Medicine, University of Oslo, P.O. Box 1111 Blindern, Oslo, NO-0317 Norway; 2 Department of Biostatistics, Institute of Basic Medical Sciences, Faculty of Medicine, University of Oslo, Oslo, Norway; 3 Institute for Studies of the Medical Profession, Oslo, Norway

**Keywords:** Veterinarians, Mental health problems, Suicidal behaviour, Personality traits, Help-seeking behaviour

## Abstract

**Background:**

Veterinarians have a relatively high prevalence of mental health problems; however, research on professional help-seeking is limited. The main purpose of the present study was to investigate the prevalence of mental health problems and professional help-seeking behaviour for such problems, and the independent factors associated with help-seeking behaviour among veterinarians in Norway.

**Method:**

This cross-sectional study included all veterinarians in Norway (response rate 75%, 70% women). Logistic regression was used to calculate odds ratios (OR) for professional help-seeking for mental health problems. Analyses were controlled for socio-demographic, individual (personality trait reality weakness, SCL-5, attitudes toward mental illness), and work-related factors (work field, job stress).

**Results:**

The prevalence of self-reported mental health problems in need of treatment was 30% (746/2494), significantly higher among women than men (36% vs. 15%). Fifty-four percent had sought professional help, women significantly more often (56%) than men (41%). Among veterinarians with serious suicidal thoughts, 50% (69/139) had sought help. Veterinarians most frequently related mental health problems to work problems (47%), women significantly more often (49%) than men (34%). Factors significantly associated with help-seeking were being female, OR = 2.11 (95% CI: 1.24–3.60), working with production animals, OR = 0.35 (0.13–0.98), public administration, OR = 2.27 (1.15–4.45), academia/research, OR = 4.78 (1.99–11.47) or ‘other’ fields, OR = 2.79 (1.23–6.32), and attitudes toward mental illness, OR = 1.32 (1.03–1.68).

**Conclusions:**

Thirty percent of veterinarians in Norway reported mental health problems in need of treatment, and only half of them had sought professional help. A low degree of help-seeking was also seen among those with serious suicidal thoughts. Being female, positive attitudes toward treatment of mental illness, working in public administration, academia/research and ‘other’ field were associated with more help-seeking, while working in production animal practice was associated with less help-seeking. Interventions to increase help-seeking behaviour for mental health problems among veterinarians are warranted.

**Supplementary Information:**

The online version contains supplementary material available at 10.1186/s12889-022-13710-y.

## Background

Several studies have found that veterinarians have a high prevalence of suicidal thoughts, anxiety symptoms, and depressive symptoms compared to the general population [1–3]. Thus, there is a need to investigate professional help-seeking behaviour for such problems [4].

In a systematic review, thirteen papers on mental health difficulties among veterinarians were reviewed. Females, younger veterinarians, and those working alone seemed more likely to experience difficulties with poor wellbeing [5]. Recent studies have found a higher prevalence of both anxiety symptoms and depression symptoms among veterinarians than in the general population [3, 6, 7].

There is limited knowledge on factors associated with professional help-seeking for mental health problems among veterinarians. A recent study found that among veterinarians with a history of mental illness, 86% had sought treatment, with no significant gender difference [3]. A study from the US showed that 59% of veterinarians with psychological distress were not receiving mental health treatment [8]. When investigating work-related factors associated with help-seeking, it is important to control for other factors that could affect help-seeking, such as age [9, 10], gender [11–13], and civil status [14]. Functional impairment has been shown to be a strong mediator of help-seeking [9, 14], indicating that people seek help when mental health problems become more severe. This is in line with a longitudinal study in physicians, where the perceived level of mental health problems predicted help-seeking [15].

To our knowledge, only one study reported help-seeking among veterinarians with suicidal behaviours. In a qualitative study, approximately half of veterinarians with a history of suicidal behaviour did not seek help because they felt guilty or ashamed [16]. In the general population, help-seeking among suicidal individuals is usually low, and several barriers, such as being male [17, 18], lack of perceived need, preference for self-management, and structural factors such as time and geographical inconvenience have been reported [17, 19]. Veterinarians and other care-giving professions may find it hard to receive help for mental health problems due to role reversal, i.e., they are used to helping others but not receiving help [15, 20]. In summary, there is a knowledge gap regarding help-seeking for mental health problems among veterinarians.

Personality traits are putative factors associated with help-seeking. Reality weakness appears to be particularly relevant; it is a deviant personality trait encompassing chronic illusions, paranoid traits, identity insecurity, and relational problems [21]. Reality weakness has previously predicted lack of help seeking among physicians, possibly because persons with this trait tend to deny their own problems, and because reality weakness is associated with severe personality disorders [15].

To the best of our knowledge, professional help-seeking behaviour among veterinarians in different fields of work has not been investigated. This could be of importance since organizational aspects differ between different fields of work, e.g., some may have more flexible work schedules as well as better accessibility to professional mental health treatment than others. Emotional demands have previously been found as a job stress factor among veterinarians in Norway [22]. The role of job stress factors has not previously been investigated with regard to help-seeking for mental health problems.

A study from the US showed that veterinarians were less likely to have positive attitudes toward mental illness than the general population [8]. Stigma associated with mental illness may lead to a reduction in help-seeking among veterinarians [23]. Recent systematic reviews in the general population and subgroups such as health professionals found that stigma is an important barrier to help-seeking [24, 25], and for health professionals, disclosure/confidentiality concerns and negative social judgements were more frequently reported than that for other groups [24].

Given this background, we investigated the following research questions:

1. What is the prevalence of self-reported mental health problems in need of treatment among veterinarians in Norway, and how many of them have sought professional help?
2. What do veterinarians regard as factors contributing to their mental health problems, and what are their overall attitudes toward mental illness?
3. What are the factors associated with actual professional help-seeking among those who consider themselves to be in need of treatment, and what are the factors associated with professional help-seeking among veterinarians with serious suicidal thoughts?

## Methods

### Sample

The sample included all veterinarians in Norway holding valid authorisation as of May 2020 (*n* = 4256), according to the Norwegian Food Safety Authority. Veterinarians were excluded for the following reasons: no residential address in Norway (*n* = 527), unknown address (*n* = 196), working abroad (*n* = 62), or deceased (*n* = 7). This resulted in an eligible sample of 3464 veterinarians.

### Questionnaire

A 12-page questionnaire, an information sheet and a prepaid envelope were distributed by mail in November 2020. The information sheet included contact information of a psychiatrist in the research group and the colleague-support network of the Norwegian Veterinary Association. Reminders were sent in January and February, 2021. Five gift cards from a sports shop were placed in a drawing as incentives to increase the response rate. An external company managed both the data collection and prize awards. Respondents returned their questionnaires in a sealed envelope, and the identities of the respondents were kept unknown to the researchers. The complete questionnaire can be found elsewhere [22].

### Instruments – dependent variable

Our main dependent variable was mental health problems in need of treatment and help-seeking for such problems, first used in physicians [26] and later a slightly altered version has been validated in the same profession [15, 27]. The veterinarians were asked the following, in line with previous studies [15, 27]: ‘If you have experienced mental health problems in the preceding year, have you sought/received help for this?’ The question had five response options from 1 to 5, indicating a higher level of perceived and/or received care with increasing scores: 1. ‘Have not had any mental health problems of significance’, 2. ‘Have not sought help despite having been in need for this’, 3. ‘Yes, consulted general practitioner’, 4. ‘Yes, consulted psychologist/psychiatrist’, 5. ‘Yes, have been admitted to psychiatric hospital’. In case of multiple responses to this question, the highest level of care was retained for the analyses.

To assess the prevalence of mental health problems in need of treatment, we dichotomised the abovementioned variables into those in need of or having sought help (response option 2–5) and those with no mental health problems of significance (response option 1). To assess the prevalence of help-seeking among those who considered themselves in need of treatment (i.e., those who answered 2–5), we computed a dichotomy with those who had actually sought help (answers 3–5 = 1) and those who had not sought help (answer 2 = 0). This dichotomy was used as the dependent variable in logistic regression analyses.

Serious suicidal thoughts were measured by a modified version of the fourth question of Paykel’s questionnaire about suicidal thoughts and attempts [28, 29]: ‘Have you ever during the last year reached the point where you seriously considered taking your life, and even made plans how you would go about doing it?’ The responses were never, hardly ever, sometimes, or often. Responses were dichotomized into never and any frequency in line with Paykel’s original work.

For those who reported having mental health problems in need of treatment, an additional question was asked: ‘To what extent do you think the following factors contributed to your difficulties?’; the factors were: 1. ‘Personal problems’, 2. ‘Family problems’, 3. ‘Social problems’, 4. ‘Work problems’, 5. ‘Other problems’. Each of the factors were rated on a categorical, ordinal five point scale ranging from ‘not at all’ (1) to ‘very much’ (5). The responses were dichotomized into ‘Not at all’, ‘A little’ and ‘Somewhat’ (0) and ‘Quite a bit’ and ‘Very much’ (1), to clearly identify factors that had a significant contribution, according to previous studies [22, 30].

### Independent variables – individual factors

The Norwegian Centre for Research Data required use of age intervals to keep the data unidentifiable. Therefore, age was reported using the following intervals: 20–25, 26–30 (…) up to 66–70 and > 70 years. In the regression analysis, we entered age as a continuous variable, since age can be considered ordinal data, and because using age categories would have generated an excessive number of effect estimates for our model. In this study, marital status was dichotomised into married/cohabitant and single/divorced/separated/widow(er).

The personality trait reality weakness was measured using the nine-item reality weakness dimension of Torgersen’s Basic Character Inventory (BCI) [31]. Each item has a dichotomous (‘agree’/’do not agree’) response, with a total sum score ranging from 0 to 9. BCI-Reality weakness is a deviant trait related to perceptions and ideations on the borderline between reality and fantasy; this dimension associates with chronic illusions, paranoid traits, and traits related to severe personality disorders [21, 32]. Examples of items in this scale are ‘I feel lonely most of the time’ and ‘Sometimes I feel I am not myself’. This measure predicts emotional disturbance in physicians, such as serious suicidal thoughts, severe depression, and lack of help-seeking [32].

Mental distress (anxiety symptoms and depressive symptoms) in the last 14 days was measured using SCL-5, a five-item version of the Symptom Check List-25 [33]. This five-item version is based on a factor analysis by Tambs and Moum [34], and contains questions about how much one is bothered by the following: 1. ‘Feeling fearful’, 2. ‘Nervousness or shakiness inside’, 3. ‘Feeling hopeless about the future’, 4. ‘Feeling blue’, 5. ‘Worrying too much about things’. Each item was measured on a scale from ‘not at all’ (1) to ‘very much’ (5). Previous studies have validated this version in medical students and physicians in Norway [35, 36].

Attitudes toward mental illness were investigated using two items originally used in public health surveys in the US [37], as well as in US veterinarians [8]. The veterinarians were asked to state their level of agreement with the following statements: 1. ‘Treatment can help people with mental illness lead normal lives’, 2. ‘People are generally caring and sympathetic to persons with mental illness’. The response categories were ‘strongly agree’, ‘somewhat agree’, ‘not sure/undecided’, ‘somewhat disagree’ and ‘strongly disagree’, coded from 1 to 5, respectively. The response scales for the two items were reversed, meaning that increasing values on the scale indicated a more positive attitude. It was then entered as continuous variables in the regression models, with increasing values on the scale indicating more positive attitudes to the two statements.

### Work-related factors

Main field of work was reported as ‘companion animal practice’, ‘production animal practice’, ‘mixed clinical practice’, ‘equine practice’, ‘aquaculture’, ‘public administration’, ‘academia/research’, ‘pensioners’, and ‘others’ [22]. Those who classified themselves as pensioners were excluded from the logistic regression analyses. In the regression analyses, ‘mixed clinical practice’ was chosen as the reference category, as this could be viewed as the most traditional veterinary work in Norway.

Job stress was measured using a modified version of Cooper’s Job Stress Questionnaire [26, 38], with minor adaptations to veterinarians’ work conditions [22]. The veterinarians were asked how much 27 different situations/factors made them stressed, with the responses reported on a five-point Likert type rating scale ranging from no stress at all (1) to a source of extreme stress (5). An exploratory factor analysis with principal component extraction and varimax rotation, including scree plot evaluation, identified three job stress factors: emotional demands (Cronbach’s alpha = 0.87), work/life balance (Cronbach’s alpha = 0.86), and fear of complaints/criticism (Cronbach’s alpha = 0.88). The job stress measure and the three sub-dimensions are explained in detail elsewhere [22].

### Statistical analysis

StataSE 16 was used for the statistical analyses. Table analyses and the χ^2^ test were used to test for gender differences. Bivariate and multivariable logistic regression models were used to estimate odds ratios (ORs) for associations between individual and work-related variables with professional help-seeking. The following factors were used as independent variables: gender, age, civil status, mental distress, reality weakness, attitudes toward mental illness, main field of work, and job stress. Initially all independent variables were analysed bivariately with the dependent variable help-seeking.

The binary variable of help-seeking (“not sought help” = 0, “sought help” = 1) was used in two logistic regression analyses, one for the group of veterinarians that considered themselves in need of treatment for mental health problems and one for veterinarians reporting serious suicidal thoughts.

For the group of veterinarians reporting mental health problems in need of treatment, we ran a multiple logistic regression analysis with all the mentioned independent variables. For veterinarians reporting serious suicidal thoughts, the independent variables in the multiple logistic regression were limited to gender, age, civil status, mental distress, reality weakness, and main field of work (due to the limited N in this subgroup).

We tested the goodness of fit of the logistic regression models, and all models were found satisfactory. The level of significance was set at 5% (*p* < 0.05). To investigate gender-specific effects, we entered two-way interaction terms between gender and the independent variables with the main effect included in the model. Interaction terms were entered one at a time.

## Results

Of 3464 eligible participants, we received 2596 responses, resulting in a response rate of 75%. The ages varied between genders, with a higher proportion of younger women, and the majority of men being older than 50 years. In total, 70% were female and 30% male, which is an accurate reflection of the actual gender distribution of veterinarians in Norway. A total of 139 veterinarians reported serious suicidal thoughts, a finding which is described in detail elsewhere [22]. See Table 1 for a description of the sample and the independent variables.

**Table 1:** Description of the independent variables in the present sample

Variable	Range of values	Frequency (%)	Mean (SD)
**Gender**			
Female		1776 (70%)	
Male		776 (30%)	
**Age**^a^			
20–30		274 (11%)	
31–40		697 (27%)	
41–50		667 (26%)	
51–60		432 (17%)	
61–70		318 (13%)	
> 70		159 (6%)	
**Marital status**			
Married/cohabiting		1962 (78%)	
Single/divorced/widow(er)		552 (22%)	
**SCL-5**	1–5		2.00 (0.98)
**Personality trait reality weakness**	0–9		1.38 (1.85)
**Main field of work**			
Companion animal practice		802 (32%)	
Public administration		402 (16%)	
Mixed clinical practice		268 (10%)	
Academia/research		202 (8%)	
Production animal practice		177 (7%)	
Aquaculture		121 (5%)	
Equine practice		102 (4%)	
Other		250 (10%)	
Pensioner		198 (8%)	
**Job stress**			
Emotional demands	1–5		1.98 (0.79)
Work/life balance	1–5		2.67 (0.97)
Fear of complaints	1–5		3.06 (1.17)
**Attitudes toward mental illness**			
Treatment help those with mental illness (somewhat agree + strongly agree)		2388 (93%)	
People are caring toward those with mental illness (somewhat agree + strongly agree)		1273 (50%)	

^a^Age was reported in five-year categories. In Table 1, age distribution is showed in 10-year categories, to improve readability

### Prevalence of mental health problems in need of treatment and professional help-seeking

The prevalence of mental health problems in need of treatment was 30% (746/2494) (95% CI 28.1%–31.8%). Overall, more women (36%, *n* = 625/1726) than men (15%, *n* = 111/730) reported mental health problems in need of treatment (χ2:107.9, *p* < 0.001).

Of the veterinarians considering themselves in need of treatment, 54% (*n* = 401/746) had sought/received professional help, and females sought help more often (56%, *n* = 350/625) than their male (41%, *n* = 45/111) colleagues (χ2: 9.06, *p* = 0.003). The prevalence of professional help-seeking was similar in all age groups (measured in ten-year intervals).

Of the veterinarians with serious suicidal thoughts, 50% (*n* = 69/139) had sought help. Significantly more women (57%, *n* = 61/108) than men (21%, *n* = 6/28) had sought help (χ2: 10.9, *p* = 0.001). Notably, 18% (*n* = 24) reported ‘no mental health problems of significance’ (response option 1).

### Self-reported factors contributing to mental health problems and attitudes toward mental illness

The most commonly reported contributing factor to mental health problems was work problems (47%, *n* = 337/712), followed by personal problems (36%, *n* = 252/706), and family problems (34%, *n* = 240/705) (Table 2). The only significant gender difference was regarding work problems, with females reporting work problems more often (49%, *n* = 296/599) than their male colleagues (34%, *n* = 36/105) (χ2: 8.2, *p* = 0.004).

**Table 2:** Factors contributing to mental health problems among veterinarians in Norway (*n* = 746)

	Not at all + A little + Somewhat N (%)	Quite a bit + Very much N (%)	Total n
Personal problems	454 (64%)	252 (36%)	706
Family problems	465 (66%)	240 (34%)	705
Social problems	608 (88%)	84 (12%)	692
Work problems^a^	375 (53%)	337 (47%)	712
Other problems	517 (79%)	139 (21%)	656

Of the veterinarians reporting mental health problems in need of treatment, there were 111 men and 625 women

^a^The only significant gender difference was in work problems

Of the 50% (*n* = 1273/2568) agreeing that people are caring toward persons with mental illness, significantly more men than women had positive attitudes (men 443/764 = 0.58 vs. women 811/1761 = 0.46, χ2: 30.3, *p* < 0.001). There was a significant difference among age groups with regard to prevalence of positive attitudes (χ2: 20.94, *p* < 0.001), with higher prevalence in the lower (20–30 years) and higher (> 70 years) age groups (measured in ten-year intervals).

### Factors associated with professional help-seeking for mental health problems

In the multivariable analyses, the significant factors associated with increased likelihood of professional help-seeking were being female, working in public administration, academia/research, and ‘other’ field of work, and positive attitudes toward treatment of mental illness. The only factor significantly associated with a lower likelihood of professional help-seeking was working in production animal practice (Table 3). Among those veterinarians with mental health problems in need of treatment, no significant interactions with gender were found.

**Table 3:** Factors associated with professional help-seeking among veterinarians with self-reported mental health problems in need of treatment (*n* = 618)

	Bivariate		Multivariable	
	OR	95% CI	OR	95% CI
Female	1.89**	1.24–2.86	2.11**	1.24–3.60
Age	1.02	0.95–1.10	1.00	0.92–1.12
Single	1.20	0.87–1.65	1.25	0.86–1.81
SCL-5	1.03	0.89–1.18	1.14	0.93–1.40
Reality weakness^a^	0.93*	0.87–0.99	0.93	0.85–1.01
**Attitudes toward mental illness**				
Treatment helps those with mental illness	1.26*	1.03–1.55	1.32*	1.03–1.68
People are caring toward those with mental illness	1.03	0.91–1.18	1.10	0.93–1.28
**Main field of work (ref. category = mixed clinical practice)**				
Companion animals	1.39	0.84–2.32	1.43	0.82–2.50
Production animals	0.33*	0.13–0.85	0.35*	0.13–0.98
Equine practice	1.67	0.72–3.87	1.75	0.70–4.36
Aquaculture	1.23	0.55–2.73	1.18	0.47–2.97
Public administration	2.31**	1.29–4.13	2.27*	1.15–4.45
Academia/research	3.44**	1.64–7.18	4.78***	1.99–11.47
Other	2.75**	1.39–5.46	2.79*	1.23–6.32
**Job stress**				
Emotional demands	1.02	0.85–1.23	1.17	0.91–1.50
Work/life-balance	0.95	0.82–1.11	0.97	0.79–1.19
Fear of complaints	0.91	0.80–1.05	0.97	0.80–1.18

^*^P < 0.05, **P < 0.01, ***P < 0.001

^a^There was a high correlation between SCL-5 and reality weakness (Pearson’s *R* = 0.6)

### Factors associated with professional help-seeking among veterinarians with serious suicidal thoughts

Being female (OR = 4.42, 95% CI 1.64–11.89) and mental distress (OR = 1.61, 95% CI 1.13–2.30) were significant bivariate factors associated with increased likelihood of professional help-seeking. There were no factors significantly associated with professional help-seeking for serious suicidal thoughts in the multivariable analyses. A full description of the analyses is found as an additional file (Additional Table 1 – help-seeking among veterinarians with serious suicidal thoughts.docx). Among those veterinarians with serious suicidal thoughts, significant interactions between gender and attitudes toward treatment of mental illness (OR = 7.15, 95% CI 1.54 to 33.27, *p* = 0.012), with clearly steeper gradients among females.

## Discussion

A major finding in this study is that 30% of veterinarians reported mental health problems in need of treatment. Only half of the veterinarians in need of treatment had sought professional help, and this also applied to veterinarians with serious suicidal thoughts. ‘Work problems’ was the most common self-reported contributing factor to mental health problems, and women reported work-related factors as contributing to mental health problems more often than men. In the multivariable analyses, being female, working in public administration, academia/research, and ‘other’ field of work, and positive attitudes toward treatment of mental illness were associated with more help-seeking, while working in production animal practice was associated with less help-seeking.

The frequency of veterinarians in Norway reporting mental health problems in need of treatment seems relatively high. The corresponding figures among physicians in Norway in their first and fourth postgraduate years were 11% and 17%, respectively [15]. However, the samples are not directly comparable due to different age groups. Higher prevalence of mental health problems among female veterinarians compared to their male colleagues is in line with the general population [39–41] and should probably not be interpreted as an occupational risk.

‘Work problems’ was the most commonly self-reported contributing factor to mental health problems among the veterinarians, especially for females; it was also the most common self-reported contributing factor for serious suicidal thoughts among veterinarians in this group [22]. These findings indicate that veterinarians perceive work problems as significant contributors to their mental health problems.

The low prevalence of professional help-seeking for mental health problems among veterinarians (54%) is comparable to physicians in Norway. A study showed that 50% and 41% of physicians had sought help for mental health problems during the first and fourth postgraduate year, respectively [15]. Among veterinarians, 9% received help from a psychologist/psychiatrist for their mental health problems. This is in line with Norwegian police, where less than 10% of those reporting anxiety symptoms or depressive symptoms or serious suicidal ideation had contacted a psychologist/psychiatrist [11]. Veterinarians in need of treatment for mental health problems had mainly sought help from their general practitioner or a psychologist/psychiatrist. In Norway, individuals are generally referred by their regular general practitioner to a psychologist/psychiatrist. This could explain the fact that there was a similar prevalence of help-seeking from both general practitioners and psychologists/psychiatrists in our sample. Accessibility to mental health specialists vary widely in Norway, and this may also have affected the results.

Female veterinarians sought help for mental health problems more often than their male colleagues. This is in line with studies on help-seeking among ambulance personnel [12] and police [11], but is in contrast to physicians, where no gender difference was found [15]. Lack of professional help-seeking for mental health problems among men is widely reported [42, 43]. Several studies have found that help-seeking is largely unaffected by age [11, 12]. Nevertheless, a recent longitudinal study among medical students found an increase in help-seeking among female students over the past 20 years [35]. In our study, the prevalence of help-seeking was not higher in the younger age groups, indicating that there was no trend toward more help-seeking among the younger veterinarians.

Mental distress was not significantly associated with professional help-seeking in the multivariable analyses. This is in line with findings among ambulance personnel, where help-seeking was independent of the level of anxiety and depressive symptoms [12]. A study among police found that less than 10% of those reporting symptoms of anxiety or depression or serious suicidal thoughts had contacted a psychologist or psychiatrist [11]. On the other side, a study among physicians in Norway found that those with higher levels of perceived mental health problems sought help more often [15]. This is also shown in the general population [9]. The finding that mental distress is associated with serious suicidal thoughts among veterinarians [22], but not professional help-seeking, may indicate that many veterinarians struggle, but do not seek help for their problems despite high symptom levels. The fact that only half of the veterinarians with self-reported mental health problems and half of those with serious suicidal thoughts sought help points to a treatment gap.

Contrary to previous research [15], the personality trait reality weakness was not a factor significantly associated with professional help-seeking in the multivariable analyses. This may be due to the high correlation between mental distress and reality weakness. Additionally, the three job stress factors were not significantly associated with professional help-seeking, neither in bivariate nor the multivariable analyses.

Working in production animal practice was associated with less professional help-seeking, and working in public administration, academia/research, and other field was associated with more help-seeking. The solitary nature of work for production animal practitioners, less flexible work schedules and geographical inconvenience could be of relevance for not seeking help [44]. Those working in public administration, research and academia may have more regulated hours and access to mental health treatment, since they more often work in urban areas. A recent study among veterinarians in the US found that those with negative attitudes concerning care and sympathy for people with mental illness were significantly more likely to be solo practitioners than being non-solo practitioners [45]. An analysis was performed to assess whether those with one or more missing items on the independent variables differed from those with no items missing. There was a slight predominance of veterinarians in public administration that had one or more missing items on the independent variables. Nevertheless, this was not suspected to have introduced any substantial bias in the model. No other trends were found in respondents with missing data.

In this survey, 93% of veterinarians somewhat or strongly agreed that treatment can help people with mental illness lead normal lives, and 50% somewhat or strongly agreed that people are generally caring and sympathetic to persons with mental illness. The corresponding figures among veterinarians in the US were 89% and 32%, respectively [8]. Positive attitudes toward treatment of mental illness was significantly associated with professional help-seeking. This contrasts with a recent review among people with mental health problems, finding that personal and perceived stigma did not have a direct effect on help-seeking [9]. However, negative attitudes toward treatment have previously been reported as a barrier to help-seeking [46], as well as poor mental health literacy [47]. Disclosure and confidentiality concerns may also be of importance for help-seeking [24, 44], with confidentiality and potential consequences for career found as significant barriers for physicians [20]. Lack of undergraduate training in psychology and ‘non-technical skills’ such as clinical communication for veterinarians may influence their attitudes toward mental illness.

The youngest and oldest age groups reported a higher prevalence of positive attitudes toward people with mental illness; however, age was not significantly associated with actual help-seeking. A recent study also showed that older age is associated with less public and self-stigma [10]. A systematic review of help-seeking interventions for anxiety, depression, and general psychological stress among young adults found that mental health literacy was effective in improving help-seeking attitudes, but not help-seeking behaviour [48]. This may partly explain the discrepancy between the relatively positive attitudes toward treatment of mental illness reported among veterinarians in Norway, and the low prevalence of actual help-seeking among those with mental health problems.

Among those with serious suicidal thoughts, the bivariate factors significantly associated with professional help-seeking were being female and the presence of mental distress, but no significant factors were found in multivariable analyses. A strong interaction between genders was found regarding attitudes toward treatment of mental illness, with women having approximately 7 times higher odds for help-seeking than men. Only one third of the veterinarians with serious suicidal thoughts had received help from a psychologist/psychiatrist, and the lack of professional help-seeking in this group is evident. Almost 20% of those reporting serious suicidal thoughts simultaneously reported ‘No significant mental health problems’ on the measure of mental health problems in need of treatment. The cause for this should be further studied qualitatively. There could be a resistance to undertake the patient role, an unwillingness to reveal any illness to their colleagues [49, 50], or there could be a low perceived need for help [46]. Reducing the barriers for seeking mental health treatment has been suggested as one measure that might reduce the risk for suicide among veterinarians [8].

To the best of our knowledge, the NORVET study is the only nationwide study of veterinarians, including all authorised veterinarians, in both clinical and non-clinical field of work. The present study is also the first to investigate factors associated with professional help-seeking behaviour among veterinarians. Major strengths of our study are the high response rate (75%) and the extensive questionnaire allowed the use of a comprehensive multivariable statistical model, controlling for several variables. Important limitations are the cross-sectional design, and the fact that generalisability of the results may be limited due to possible differences in organization of work life in different countries. Nevertheless, we believe the findings are representative of veterinarians in Northern Europe. Further studies should aim to include multiple data sources, for example from health registers. The study was conducted during the coronavirus pandemic, which may have affected the results, since potential effects of the pandemic (e.g., redundancy, economic effects) were not accounted for. Another limitation is that the dependent variable was based on a single-item, self-reporting measure, although there were several response categories with respect to professional help-seeking. Due to social desirability, this may have led to under-reporting and type II errors (false negatives). Veterinarians may have limited competency in assessing their own mental health and may not be aware that they actually have symptoms of mental illness, which also could lead to under-reporting. Additionally, since the measure did not define mental health problems, we do not know what kind of mental health problems comprise the dependent variable. Another limitation is the disparity in the referred time span measured by mental health problems (12 months) and mental distress (past 2 weeks).

## Conclusions

Thirty percent of veterinarians in Norway reported mental health problems in need of treatment, and only half of them had sought professional help. First, continued research on veterinarians and mental health is of importance, both longitudinal studies which can elaborate further on possible causal factors for mental health problems, and qualitative studies to achieve a deeper understanding of factors impacting mental health. Second, interventions on different levels should be assessed, for instance inclusion of mental health literacy in the curriculum and establishing a low-threshold, easily accessible help service for struggling veterinarians, which has been available for physicians in Norway for decades [36]. Such interventions may decrease stigma and increase help-seeking behaviour for mental health problems among veterinarians in Norway.

## Supplementary Information

**Additional file 1: Table 1.** Factors associated with professional help-seeking among veterinarians with serious suicidal thoughts (n=127).

## Data Availability

Data are available upon reasonable request. Requests can be directed to the corresponding author.
