# Supplementary material for: Professional help-seeking behaviour for mental health problems among veterinarians in Norway: a nationwide, cross-sectional study (The NORVET study)
Source: BMC Public Health. 2022 Jul 7;22:1308. doi: 10.1186/s12889-022-13710-y (PMC9263054; doi:10.1186/s12889-022-13710-y)
Supplement: Supplementary file 1 — Additional file 1: Table 1. Factors associated with professional help-seeking among veterinarians with serious suicidal thoughts (n=127). [file 12889_2022_13710_MOESM1_ESM.docx]

Supplementary table 1 – Factors associated with professional help-seeking among veterinarians with serious suicidal thoughts (n=127)

|  | Bivariate | | Multivariable | |
| --- | --- | --- | --- | --- |
|  | OR | 95 % CI | OR | 95 % CI |
| Female | 4.42** | 1.64–11.89 | 2.93 | 0.91–9.40 |
| Age | 0.89 | 0.76–1.04 | 0.91 | 0.73–1.14 |
| Single | 1.89 | 0.93–3.87 | 1.41 | 0.61–3.29 |
| SCL-5 | 1.61** | 1.13–2.30 | 1.56 | 0.99–2.46 |
| Reality weakness^1^ | 1.08 | 0.92–1.26 | 0.99 | 0.80–1.21 |
| **Attitudes toward mental illness** |  |  |  |  |
| Treatment helps those with mental illness | 1.21 | 0.81–1.81 | 1.44 | 0.90–2.31 |
| People are caring toward those with mental illness | 0.92 | 0.66–1.27 | 0.97 | 0.65–1.43 |
| **Main field of work (ref. category= mixed clinical practice)** |  |  |  |  |
| Companion animals | 0.83 | 0.24–2.79 | 0.78 | 0.20–3.04 |
| Production animals | 0.19 | 0.03–1.25 | 0.26 | 0.03–2.01 |
| Equine practice | 0.43 | 0.06–3.22 | 0.61 | 0.06–6.23 |
| Aquaculture | 0.86 | 0.12–5.94 | 1.15 | 0.14–9.74 |
| Public administration | 1.71 | 0.41–7.08 | 3.06 | 0.60–15.60 |
| Academia/research | 1.50 | 0.29–7.75 | 1.89 | 0.30–11.74 |
| Other | 1.29 | 0.24–6.83 | 2.03 | 0.27–14.98 |

**P<0.01

^1^ There was a high correlation between SCL-5 and reality weakness (Pearson’s R=0.6).
